# Hydration and health at ages 40–70 years in Salzburg Austria is associated with a median total water intake over 40 mL/kg including at least 1 L/d plain drinking water

**DOI:** 10.3389/fpubh.2025.1668981

**Published:** 2025-11-07

**Authors:** Jodi D. Stookey, Patrick B. Langthaler, Thomas K. Felder, Vanessa N. Frey, Antje van der Zee-Neuen, Karin Schindler, Ludmilla Kedenko, Bernhard Iglseder, Eugen Trinka, Florian Lang, Dieter Häussinger, Markus Ritter, Bernhard Paulweber

**Affiliations:** 1 Water & Hydration Translational Epidemiological Research (WAHTER) LLC, San Francisco, CA, United States; 2 Center for Physiology, Pathophysiology and Biophysics, Institute of Physiology and Pathophysiology, Paracelsus Medical University, Salzburg, Austria; 3 Department of Neurology, Christian Doppler University Hospital, Paracelsus Medical University and Centre for Cognitive Neuroscience, Member of the European Reference Network EpiCARE, Salzburg, Austria; 4 Department of Laboratory Medicine, Paracelsus Medical University, Salzburg, Austria; 5 Institute of Pharmacy, Paracelsus Medical University, Salzburg, Austria; 6 Gastein Research Institute, Paracelsus Medical University, Salzburg, Austria; 7 Division of Endocrinology and Metabolism, Medical University Vienna, Vienna, Austria; 8 Department of Internal Medicine I, St. Johanns University Hospital, Paracelsus Medical University, Salzburg, Austria; 9 Department of Geriatrics, Christian Doppler University Hospital, Paracelsus Medical University, Salzburg, Austria; 10 Neuroscience Institute, Center for Cognitive Neuroscience, Christian Doppler University Hospital, Paracelsus Medical University, Salzburg, Austria; 11 Karl Landsteiner Institute of Neurorehabilitation and Space Neurology, Salzburg, Austria; 12 Department of Physiology, Eberhard Karls University, Tuebingen, Germany; 13 Clinic for Gastroenterology, Hepatology, and Infectious Diseases, Medical Faculty, Heinrich Heine University Düsseldorf, Düsseldorf, Germany; 14 Ludwig Boltzmann Institute for Arthritis and Rehabilitation, Paracelsus Medical University, Salzburg, Austria; 15 Kathmandu University School of Medical Sciences, Dhulikhel, Nepal

**Keywords:** drinking water, water intake, hydration, adequate intake, Austria

## Abstract

**Introduction:**

To address longstanding questions about how much plain water to drink for hydration and long-term health, this study described the plain water intake (PWI) of people without chronic health conditions (CHC), at ages 40–70 years, who met hydration criteria (Healthy+Hydrated).

**Methods:**

Community-dwelling participants in the population-based Paracelsus 10,000 study in Salzburg, Austria (*n* = 5,817, 40–70 years), completed the EPIC diet questionnaire, blood and urine collection, and clinical assessment for CHC, including obesity, diabetes, hypertension, metabolic syndrome, cancer, liver, digestive tract, lung, kidney, cardiovascular and cerebrovascular disorders. Participants with serum tonicity 285–294 mOsmol/L and urine specific gravity (USPG) < 1.013 were classified as Hydrated. Cross-sectional analyses described the PWI of Healthy+Hydrated adults, compared to groups not meeting criteria for hydration (Healthy+Not Hydrated), health (CHC+Hydrated), or both (CHC+Not Hydrated), and relative to body weight and total water intake (TWI).

**Results:**

For Healthy+Hydrated women, the median PWI and TWI were 1.5 L/d (22 mL/kg) and 2.9 L/d (45 mL/kg), respectively. For Healthy+Hydrated men, the median PWI and TWI were 1.3 L/d (17 mL/kg) and 3.0 L/d (40 mL/kg). None of the Healthy+Hydrated reported zero PWI. In gender-specific Poisson models, the Healthy+Hydrated group had significantly lower relative risk of PWI < 20 mL/kg AND TWI < 45 mL/kg than each of the CHC+Hydrated, Healthy+Not Hydrated, and CHC+Not Hydrated groups. For Healthy+Hydrated participants with >60% of TWI from PWI, PWI ranged between 20 and 45 mL/kg/d.

**Conclusion:**

In the Paracelsus 10,000 study population, hydration and health at ages 40–70 years was associated with a median PWI of at least 1 L/d.

## 1 Introduction

Data are needed to inform recommendations about how much plain water to drink for long-term health. In countries around the world, available water intake recommendations technically only address how much water to ingest from the sum of all foods and/or beverages, of any type, and only aim to reduce the risks of short-term health problems (1). Health professionals caution against drawing inferences about drinking water from total water intake (TWI) recommendations, citing no specific evidence about plain water intake (PWI) (2, 3).

TWI recommendations of 2.0–2.5 L/d roughly equate to about eight glasses of PWI in health education materials [e.g., see Ref. (4, 5)]. Although liters or glasses of PWI are consistent with health authority guidance that water from *any* source satisfies water requirements (i.e., including drinking water as sole source) (6, 7), the messaging runs counter to daily life experience where PWI is not the sole source of TWI. For two decades, health professionals have warned that people who consume water from foods and various beverages, do not need to drink 2 L/d plain water (2, 3). Yamada et al. (3), for example, contend that “objective evidence” would be needed to back advice about how much PWI the average person needs. It remains to be determined what volume of PWI is adequate, given a range of circumstances, for example, when PWI accounts for a small fraction of TWI vs. the majority of TWI.

It is unknown how much PWI is needed for long-term health. Available Adequate Intake (AI) recommendations for water [e.g., see Ref. (6, 7)] are explicitly designed to prevent short-term effects of dehydration, only, such as impaired thermoregulation, cardiovascular function, cognitive, and physical work performance. The recommendations cannot be used as surrogate recommendations for long-term health, because they were derived from datasets that include people who already have chronic health conditions (CHC), and thus may underestimate the level of water intake required to prevent incident chronic conditions. CHC, including obesity, insulin resistance, diabetes, metabolic syndrome, cancer, and decline of cognitive function, which are prevalent in population-based data, are associated with underhydration and/or lower water intake [e.g., see Ref. (8–10)].

To resolve longstanding questions about how much plain water to *drink*, this study aimed to contribute information about the PWI of adults, in Austria, who both met hydration criteria and had no chronic health condition at ages 40–70 years. Gender-specific PWI estimates for this group are described (1) relative to groups that do not meet chronic health and/or hydration criteria; (2) relative to TWI, given the *ad-libitum* diet and daily life conditions of the study population; and (3) for the special condition when PWI accounts for the majority (>60%) of TWI.

## 2 Materials and methods

### 2.1 Study design

This cross-sectional analysis used baseline data collected for the population-based Paracelsus 10,000 study in Salzburg, Austria (11). This longitudinal cohort study aims to follow 10,000 adults, over successive periods of 5–7 years, to learn about lifestyle and genetic effects on age-related chronic disease. The goals, design, and methods are described in detail elsewhere (11). The protocol for baseline data collection involved one in-person clinic visit for collection of blood and urine between 7:30 and 10:30 a.m., after at least 10 h of fasting, clinical measurements, and survey administration. This study was conducted according to the guidelines laid down in the Declaration of Helsinki and all procedures involving research study participants were approved by the Ethics Committee of the State of Salzburg (415-E/1521/3-2012). Written informed consent was obtained from all study participants. Recruitment and data collection were completed on a rolling basis between 2013 and 2020.

### 2.2 Study population

A balanced sample of women and men, ages 40–70 years, with oversampling of individuals ages 50–59 years, was recruited by repeated gender- and age-group stratified random sampling of the population registry of the metropolitan area of Salzburg. A total of 56,595 individuals were invited to participate, of whom 10,060 completed at least partial baseline assessments. Participation was voluntary and without financial compensation. The present analysis focused on individuals with complete data for hydration biomarkers, health conditions, as well as dietary intake (*n* = 5,817). Appendix 1 describes the participant recruitment and completion of data collection.

### 2.3 Laboratory measures

The spot urine and blood samples were analyzed by a laboratory that is certified according to ISO 9001:2015 and voluntarily follows the requirements of ISO 15189:2012 (11). Urine specific gravity (USPG) and urine albumin/creatinine were determined by Cobas 6000™ and Cobas U 601™ fully automated analyzers (Roche Diagnostics GmbH). Serum electrolytes (mEq/L), glucose (mg/dL), insulin (μIU/L), HbA1c (%), triglycerides (mg/dL), total cholesterol, LDL- and HDL-cholesterol (mg/dL) were determined using Cobas 6000™ and Cobas Integra 400 Plus Chemistry Analyzers™ (Roche Diagnostics GmbH). The Homeostatic Model Assessment for Insulin Resistance (HOMA-IR) was calculated as: [fasting insulin (mU/L)] × [fasting glucose (mg/dL)]/405 (12). The glomerular filtration rate (eGFR) was estimated using the Modification of Diet in Renal Disease (MDRD) Study equation (mL × 1.73 m^2^/min): 175 × [serum creatinine (mg/dL)] – 1.154 × (age)−0.203 × (0.742 for women) (13). Serum tonicity (mOsmol/L) was calculated using the formula by Matz: Eosm = 2 ^*^ [sodium (mEq/L)+ potassium (mEq/L)]+[glucose (mg/dL)/18] (14).

#### 2.3.1 Hydration biomarkers

Study participants were classified as “Hydrated” if serum tonicity was greater than or equal to 285 mOsmol/L and less than 295 mOsmol/L and urine specific gravity (USPG) was below 1.013 g/mL, consistent with published cutoffs (8, 15). Individuals who did not meet both the serum tonicity and USPG criteria were classified as “Not Hydrated.” To check for bias related to missing USPG data for 1,698 participants, sensitivity analyses were done with hydration status defined using urine creatinine less than 89 mg/dL instead of USPG below 1.013 g/mL. Over 90% of study participants with USPG below 1.013 g/mL had a urine creatinine value below 89 mg/dL.

### 2.4 Clinical measures

Abdominal waist circumference (cm), body height (m), and weight (kg) were measured at the clinic. Body mass index (BMI) was calculated as weight divided by height squared. Blood pressure was measured three times on the left arm by a dual-cuff blood pressure monitor (Watch BP office AFIB™, Microlife AG Swiss Corporation, Switzerland) in a sitting position, with a previous resting period of each 60 s, and averaged to calculate mean systolic blood pressure (mmHg) and diastolic blood pressure (mmHg). The total plaque area of the carotid arteries was measured bilaterally by ultrasound examination (CardioHealth^®^ Station, Panasonic Healthcare Diagnostics, U.S.). Trained clinical staff interviewed study participants about their medical history and medication use.

Based on laboratory test results, clinical measurements, and/or responses to the medical history interview, participants were classified as having evidence of disorders involving the: liver (cirrhosis or chronic hepatitis), digestive tract (Crohn's disease or colitis ulcerosa), lung (COPD, asthma, pulmonary embolism), kidney (eGFR < 60, urine albumin/creatinine ≥30, glomerular nephritis), cardiovascular, or cerebrovascular system (coronary artery disease, peripheral arterial disease, abdominal aortic aneurysm, atrial fibrillation, chronic heart failure, carotid artery stenosis (>50%), and stroke), underweight (BMI < 18.5), obesity (BMI ≥ 30.0), diabetes (Diabetes Type I, Diabetes Type II, fasting glucose ≥110 mg/dL, antidiabetic medication, and HOMA-IR ≥ 2.5), hypertension (mean systolic blood pressure ≥140, mean diastolic blood pressure ≥90, or antihypertension medication), metabolic syndrome, or cancer. Metabolic syndrome was defined using the criteria described by Grundy et al. (16), three or more of the following conditions: elevated abdominal circumference (≥89 cm for women, ≥102 cm for men); elevated serum triglycerides (≥150 mg/dL or on triglyceride lowering drug therapy); Low serum HDL cholesterol (< 50 mg/dL for women, < 40 for men); elevated blood pressure (mean SBP ≥130, mean DBP ≥85, or on antihypertensive drug therapy); elevated serum glucose (fasting glucose ≥100 or on antidiabetic drug therapy).

#### 2.4.1 Chronic health condition

Individuals with obesity, diabetes, hypertension, metabolic syndrome, cancer or evidence of any disorder of the liver, digestive tract, lung, kidney, cardiovascular or cerebrovascular system were classified as having a chronic health condition (CHC). Individuals with none of the listed conditions were classified as “Healthy.”

### 2.5 Dietary intake

Usual mean daily intakes, over the last 12 months, of total energy, carbohydrates, protein, sodium, potassium, chloride, phosphorus, total water, and drinking water were estimated from participant responses on the European Prospective Investigation into Cancer and Nutrition (EPIC) questionnaire, which was validated for use in Germany (17). The potential renal solute load (PRSL) of the diet was calculated as: PRSL (mOsm/d) = [urea nitrogen (mmol/d)]+[sodium (mmol/d)]+ [potassium (mmol/d)]+[chloride (mmol/d)]+[phosphorus (mmol/d)] (18), with urea nitrogen estimated as ([protein (g)]/6.25) ^*^ 1,000/28) (19). Plain water intake (PWI) and total water intake (TWI) were expressed in units of L/d as well as mL/kg of body weight. A dichotomous variable classified participant water intake as lower (PWI < 20 mL/kg/d AND TWI < 45 mL/kg/d) or higher (PWI ≥ 20 mL/kg/d OR TWI ≥ 45 mL/kg/d). To gauge PWI relative to other sources of fluid in the diet, PWI was expressed as a proportion of TWI. The pattern of TWI composition was described in bivariate terms, using only PWI and TWI, without distinguishing water from food or water from other beverages, because the latter sources are essentially isotonic or hypertonic relative to blood (osmolality >285 mOsmol/kg) (20), while drinking water is hypotonic (osmolality below 20 mOsmol/kg).

### 2.6 Determinants of water requirements

Place-based factors, such as altitude and water quality, which are recognized determinants of water requirements (7), were assumed homogeneous, because the Paracelsus 10,000 cohort was drawn from a single metropolitan area. In addition to age, gender, body size, health condition, and PRSL, data were collected about medication use, cold or warm season timing of data collection, physical activity level, and smoking. Participants were asked for current use of antidiabetic-, antihypertensive- and lipid-lowering drugs. They answered validated questions (21) from the Physical Activity Part of the EPIC Study about exercise activities not related to transportation or work, such as sports, cycling, and high intensity chores. The weekly hours spent doing the activities in the last 7 days were estimated by multiplying the reported number of hours per day by 7. Lower vs. higher physical activity was defined as no or < 10 h of reported physical activity. Participants were considered current smokers if they reported any current use of cigarettes.

### 2.7 Statistical analysis

Study participants were classified into four groups based on chronic health condition (CHC or Healthy) and hydration (Hydrated or Not Hydrated). The groups were compared with respect to determinants of water requirements, using *t*-test with Welch correction and the Healthy+Hydrated group identified as a reference.

Gender-specific Poisson models with robust variance estimation tested for greater relative risk of lower water intake in the CHC+Not Hydrated, CHC+Hydrated, and/or Healthy+Not Hydrated groups, compared to the Healthy+Hydrated group. To check for risk of lower water intake, given the real-life distribution of water requirements in the study population, the initial unadjusted models did not control for determinants of water requirements. Results from unadjusted models were compared with results from models that adjusted for proxies of water requirements to explore if lower water intake in a group might be explained by lower water requirements in the group. Adjusted models controlled for antihypertensive medication, hours of exercise per week (categorical, see above), renal solute load (below vs. above gender-specific median), hypertension, kidney disorder, smoking status, and the season at which measurements were taken (May–October vs. November–April). A final model additionally adjusted for BMI outside the healthy 18.5–25 kg/m^2^ range, to separately consider differences in body size as potential explanation of between-group differences in water intake. In sensitivity analyses, all Poisson models were re-fit using the hydration classification based on urinary creatinine concentration instead of specific urine gravity.

The gender-specific bivariate distributions of PWI and TWI were described for the Healthy+Hydrated group. The gender-specific median PWI and TWI for the Healthy+Hydrated group were estimated, with TWI source(s) unspecified, i.e., as observed, given the conditions of daily life and usual diet in metropolitan Salzburg, Austria. The gender-specific median PWI and TWI were also estimated for the subgroup of Healthy+Hydrated with over 60% of TWI from PWI, i.e., conditions when PWI is the majority source of TWI. All statistical analyses were done using the statistical software R (version 4.2.2; https://www.R-project.org/).

## 3 Results

### 3.1 Characteristics of the study population

All study participants were between the ages of 40–70 years. Table 1 describes the mean (SD) body size, hours of exercise, dietary intake, PRSL, and eGFR of the study population by gender. Women (*n* = 2,988) ranged in body weight from 38 to 160 kg. Men in the study population (*n* = 2,829) ranged in body weight from 51 to 183 kg. Over 70% of women and men reported less than 10 h/week of physical activity. Women reported a typical mean daily energy and sodium intake of approximately 2,000 kcal/d and 2,000 mg/d, respectively. Men reported approximately 2,500 kcal/d and 2,500 mg/d sodium. The median TWI was 2.7 L/d (39.9 mL/kg/d) and 2.8 L/d (32.4 mL/kg) for women and men in the Paracelsus 10,000 cohort. The median PWI was 1.1 L/d (17 mL/kg) and 0.7 L/d (10 mL/kg/d) for women and men, respectively. Appendix 2 provides information about other sources of water intake, for context, though this analysis focused only on PWI.

**Table 1:** Mean (SD) characteristics of participants in the Paracelsus 10,000 cohort by gender, health, and hydration classification.

**Participant characteristics**	**All participants (*****n*** = **2,988)**		**Healthy**+**Hydrated (*****n*** = **251)**		**Healthy**+**Not Hydrated (*****n*** = **1,083)**		**CHC**+**Hydrated (*****n*** = **192)**		**CHC**+**Not Hydrated (*****n*** = **1,462)**	
	**Mean**	**SD**	**Mean**	**SD**	**Mean**	**SD**	**Mean**	**SD**	**Mean**	**SD**
**Women**										
**Water requirements**										
Height, cm	165.3	6	165.9	6.3	165.8	5.9	165.3	6.1	164.9	6.1^*^
Weight, kg	69.3	13.7	63.2	8.7	64.2	8.2	71	14.4^*^	73.9	15.8^*^
BMI, kg/m^2^	25.4	4.9	22.9	2.6	23.4	2.6^*^	26	5.3^*^	27.2	5.6^*^
Exercise, h/week^†^	7.3	6.4	7	6.1	7.5	6.4	6.1	4.9	7.3	6.7
Total energy intake, kcal	2,007	661	2,033	664	1,979	609	1,993	685	2,026	692
Carbohydrate intake, g/d	194.4	69.8	193.2	66	191.9	63.8	193.1	72.7	196.6	74.2
Protein intake, g/d	66.2	23.6	67.2	27.7	64.9	21	65.3	22.9	67	24.7
Sodium intake, mg/d	1,935	634	1,964	669	1,902	597	1,931	595	1,955	658
PRSL, mOsm/d	655.8	213	666	238	645.2	193.4	649.6	206.8	662.8	222.8
eGFR, mL × 1.73 m^2^/min	80.6	14.3	83.2	12.2	81.5	12.4	81.2	14.6	79.3	15.8^*^
Total water intake, L/d	2.8	0.9	2.9	0.9	2.8	0.8^*^	2.8	0.8	2.8	0.9^*^
**Water intake**										
Drinking water, L/d	1.2	0.6	1.3	0.6	1.2	0.6^*^	1.3	0.6	1.2	0.6^*^
Total water intake, mL/kg/d	41.7	15.1	47.2	15.1	43.9	14.2^*^	40.9	13.1^*^	39.3	15.5^*^
Drinking water, mL/kg/d	17.6	9.6	20.5	10.1	18.5	10^*^	18.4	9.4^*^	16.4	9.2^*^
**Hydration**										
Serum sodium, mmol/L	141.3	1.9	139.1	1	141.6	1.7^*^	138.9	1.2	141.8	1.8^*^
Serum tonicity, mOsm/kgH_2_O	296.4	4	291.5	2	296.6	3.5^*^	291.4	2.2	297.7	3.8^*^
Urine specific gravity	1.014	0.007	1.007	0.003	1.014	0.007^*^	1.007	0.003	1.015	0.007^*^
**Health**										
Albumin/Creatinine ratio	8.4	37.4	5.4	5.5	4.8	4.8	10	16^*^	11.3	52.8^*^
Abdominal circumference, cm	87.6	12.2	82.5	8	82.5	8	89.6	12.3^*^	92	13.4^*^
Fasting glucose, mg/dL	91.1	12.7	86.5	6.5	87	7.2	90.2	9.9^*^	95.1	15.5^*^
**Health**										
Fasting insulin, μIU/mL	8.4	6.7	5.7	2	6	2.1^*^	8.6	4.5^*^	10.6	8.6^*^
HOMA-IR	1.98	2.36	1.23	0.47	1.29	0.49^*^	1.96	1.16^*^	2.61	3.18^*^
SBP, mmHg	125.4	17.1	116.3	10.3	117.1	10.1	130.8	17.5^*^	132.5	18.5^*^
DBP, mmHg	78.9	9.7	74.8	6.8	74.6	7	82.1	10^*^	82.3	10.3^*^
Trigylcerides mg/dL	97	60.7	74.4	27.2	81.1	30.2^*^	104.3	65^*^	111.6	75.4^*^
HDL-Cholesterol, mg/dL	71.4	17.5	78.5	17.1	74.6	16.1^*^	70.7	18.2^*^	67.8	17.7^*^
**Participant characteristics**	**All participants (*****n*** = **2,829)**		**Healthy**+**Hydrated (*****n*** = **66)**		**Healthy**+**Not Hydrated (*****n*** = **759)**		**CHC**+**Hydrated (*****n*** = **118)**		**CHC**+**Not Hydrated (*****n*** = **1,886)**	
	**Mean**	**SD**	**Mean**	**SD**	**Mean**	**SD**	**Mean**	**SD**	**Mean**	**SD**
**Men**										
**Water requirements**										
Height, cm	178.1	6.6	177.1	5.5	178.4	6.5	177	6.3	178	6.6
Weight, kg	85.9	14.1	75.1	7.4	78.7	9.2^*^	86	14.5^*^	89.1	14.7^*^
BMI, kg/m^2^	27.1	4.1	24	2.3	24.7	2.3^*^	27.4	4.1^*^	28.1	4.3^*^
Exercise, h/week^†^	7.2	6.5	7.5	4.3	7.8	6.5	6.6	5.0	7.0	6.7
Total energy intake, kcal	2,585	833.5	2,546	747	2,595	794	2,597	927	2,582	846
Carbohydrate intake, g/d	247.9	90.5	252.3	81.7	251.2	87.5	244.0	107.7	246.6	90.9
Protein intake, g/d	87.6	30.3	83.2	23.8	87.8	29.0	88.4	36.0	87.6	30.6
Sodium intake, mg/d	2,533	833.9	2,387	727	2,528	809	2,556	1,003	2,539	835.8
PRSL, mOsm/d	850.6	272.4	811.1	225.1	852.1	260.3	859.9	323.5	850.9	275.3
eGFR, mL × 1.73 m^2^/min	83.8	15.5	84.3	13	85.1	12.4	86.4	14.3	83.2	16.7
**Water intake**										
Total water intake, L/d	2.9	1.0	3.1	1.0	2.9	1.0	3.2	1.2	2.9	1.1^*^
Drinking water, L/d	1.0	0.6	1.2	0.6	1.0	0.6^*^	1.1	0.6	0.9	0.6^*^
**Water intake**										
Total water intake, mL/kg/d	34.5	13.5	42.2	14.6	37.4	13.9^*^	37.5	15.0^*^	32.9	12.9^*^
Drinking water, mL/kg/d	11.5	7.7	16.4	9.2	12.8	7.9^*^	13.2	8.2^*^	10.8	7.3^*^
**Hydration**										
Serum sodium, mmol/L	141.3	1.9	139.1	1.1	141.6	1.7^*^	138.8	1.2	141.5	1.8^*^
Serum tonicity, mOsm/kgH_2_O	296.9	3.7	291.9	2.1	297	3.4^*^	291.6	2.2	297.3	3.6^*^
Urine specific gravity	1.018	0.007	1.008	0.003	1.018	0.007^*^	1.008	0.003	1.019	0.006^*^
**Health**										
Albumin/Creatinine ratio	7.7	26.9	3.5	3.6	3.0	3.3	5.8	12.1	9.9	32.6^*^
Abdominal circumference, cm	98.3	11.5	88.3	6.8	91.4	7.5^*^	99.4	12.0^*^	101.4	11.5^*^
Fasting glucose, mg/dL	97.9	16.6	90.5	8.4	91.1	7	95.9	11.6^*^	101	18.7^*^
Fasting insulin, μIU/mL	10.8	8.6	5.4	2.0	6.3	2.1^*^	10.6	6.7^*^	12.9	9.6^*^
HOMA-IR	2.74	2.67	1.22	0.45	1.42	0.5^*^	2.54	1.73^*^	3.34	3.04^*^
SBP, mmHg	132.8	15.2	122.2	7.3	122.4	8.5	136.5	15.6^*^	137.2	15.3^*^
DBP, mmHg	85.2	9.3	79.1	4.8	79.1	6.1	87.3	10.2^*^	87.7	9.2^*^
Trigylcerides mg/dL	129.9	82.2	94.3	43.2	98.4	45.5	153.4	109.6^*^	142.4	88.6^*^
HDL-Cholesterol, mg/dL	55.5	14.2	62.6	16.5	59.7	13.5	55.3	14.2^*^	53.6	14^*^

Healthy: none of the specified chronic health conditions (obesity, diabetes, hypertension, metabolic syndrome, cancer or evidence of any disorder of the liver, digestive tract, lung, kidney, or cardiovascular system); HOMA-IR: Homeostatic Model Assessment for Insulin Resistance calculated as: [fasting insulin (mU/L)] x [fasting glucose (mg/dL)]/405; SBP: Systolic blood pressure; DBP: Diastolic blood pressure; Hydrated: Serum tonicity ≥285 and ≤ 294 **AND** specific urine gravity < 1.013; PRSL: Potential renal solute load from diet; eGFR: estimated glomerular filtration rate.

^*^Significantly different mean (SD) compared to the corresponding value for the Healthy+Hydrated group, *p* < 0.05.

^†^Self-reported leisure exercise of less than 10 h/week was used to index lower (vs. higher) physical activity.

### 3.2 Health and hydration classification

Chronic health conditions were prevalent in this non-acutely ill study population. One or more CHCs were observed for 55% of women and 71% of men. Obesity, diabetes (pre-diabetes, diabetes, or insulin resistance), and hypertension affected 15%, 22% and 29% of the women and 20%, 39% and 50% of the men, respectively (Appendix 3). The mean eGFR was 80.6 mL/min^*^1.73 m^2^ for women and 83.8 mL/min^*^1.73 m^2^ for men. The eGFR was below 90, i.e., suggestive of early-stage chronic kidney disease, for 77% of women and 69% of men.

**Figure 1:**
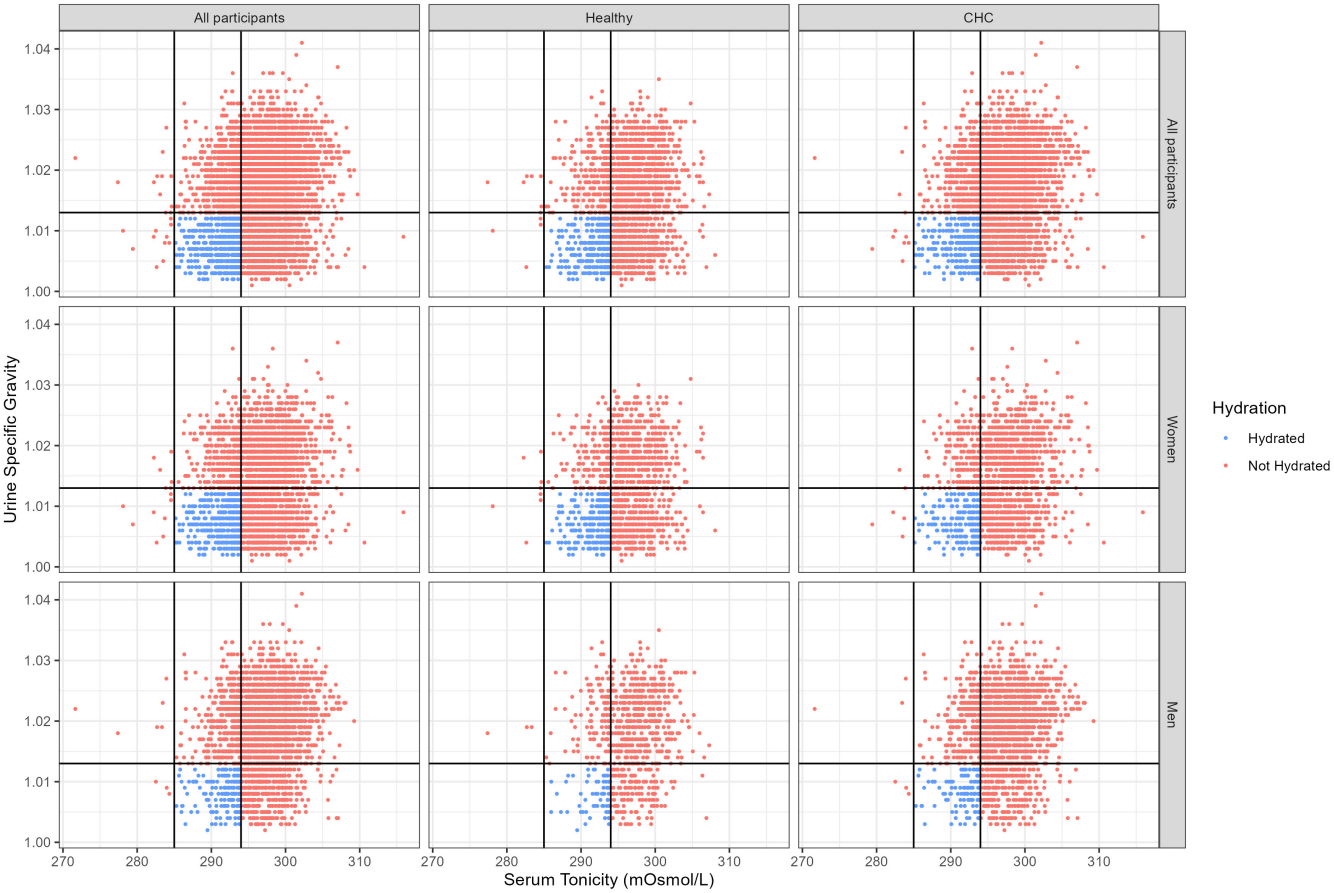
Bivariate distribution of serum tonicity and urine specific gravity for the Paracelsus 10,000 study cohort by chronic health condition and hydration classification. Healthy: none of the specified chronic health conditions (obesity, diabetes, hypertension, metabolic syndrome, cancer or evidence of any disorder of the liver, digestive tract, lung, kidney, or cardiovascular system); CHC: one or more chronic health condition; Hydrated: serum tonicity ≥285 and ≤ 294 AND specific urine gravity < 1.013.

Serum tonicity ranged from 272 to 316 mOsmol/L. Urine specific gravity ranged from 1.001 to 1.041. Serum tonicity was between 285 and 295 mOsmol/L for 35% of the women and 30% of the men. Urine specific gravity was below 1.013 for 45% of the women and 22% of the men. Only 15% of women and 7% of men met both hydration criteria. Many participants who met the serum tonicity criterion did so in conjunction with a urine specific gravity of 1.013 or higher (Figure 1).

Of 2,988 women and 2,829 men, only 251 women and 66 men were classified as free of chronic health conditions and hydrated (Healthy+Hydrated; see Table 1). Over 85% of the healthy participants did not meet the hydration criteria. Among women, the Healthy+Hydrated group was significantly taller than the CHC+Not Hydrated group. For both genders, the Healthy+Hydrated group had significantly lower body weight than the groups with CHC. Men in the Healthy+Hydrated group also had lower body weight then men in the Healthy+Not Hydrated group.

### 3.3 Relative risk of lower vs. higher water intake

The Healthy+Hydrated group was significantly less likely to report TWI below 45 mL/kg and PWI below 20 mL/kg than the other groups. The relative risk (RR) of reporting lower water intake was 20%−40% higher for participants who either did not meet hydration criteria (Healthy+Not Hydrated) or did not meet health criteria (CHC+Hydrated), depending on the reported gender and group (see Table 2 for each gender-and group-specific result). The RR of reporting lower water intake was over 50% higher for participants who neither met the CHC nor hydration criteria (CHC+Not Hydrated). In the CHC+Not Hydrated group, 61% of women and 79% of men reported lower water intake. The corresponding values for the Healthy+Hydrated group were 39 and 52%.

**Table 2:** Relative risk of lower water intake associated with chronic health condition and hydration classification.

**Participant characteristics**	**Health condition**	**Hydration classification**	**Level of water intake**			**Relative risk of having lower water intake**					
			**Higher**	**Lower**		**Unadjusted**		**Model 1**		**Model 2**	
			***n***	***n***	**Row %**	**RR (95% CI)**	***p*** **-Value**	**RR (95%CI)**	***p*** **-Value**	**RR (95% CI)**	***p*** **-Value**
Women	Healthy	Hydrated	97	154	38.6	1.0	–	1.0	–	1.0	–
	Healthy	Not Hydrated	524	559	48.4	1.25 (1.06, 1.48)	0.009	1.26 (1.06, 1.48)	0.007	1.26 (1.06, 1.48)	0.007
	CHC	Hydrated	95	97	49.5	1.28 (1.04, 1.58)	0.022	1.21 (0.98, 1.50)	0.077	1.13 (0.91, 1.39)	0.276
	CHC	Not Hydrated	886	576	60.6	1.57 (1.33, 1.84)	< 0.001	1.49 (1.26, 1.76)	< 0.001	1.36 (1.15, 1.62)	< 0.001
Men	Healthy	Hydrated	34	32	51.5	1.0	–	1.0	–	1.0	–
	Healthy	Not Hydrated	533	226	70.2	1.36 (1.07, 1.73)	0.011	1.35 (1.07, 1.71)	0.011	1.35 (1.07, 1.71)	0.011
	CHC	Hydrated	83	35	70.3	1.37 (1.05, 1.77)	0.020	1.34 (1.04, 1.74)	0.026	1.30 (1.01, 1.68)	0.044
	CHC	Not Hydrated	1,505	381	78.8	1.55 (1.22, 1.96)	< 0.001	1.53 (1.21, 1.93)	< 0.001	1.46 (1.16, 1.85)	0.001

Lower water intake: TWI < 45 mL/kg **AND** PWI < 20 mL/kg; Higher water intake: TWI ≥ 45 mL/kg **OR** PWI ≥ 20 mL/kg; TWI, total water intake; PWI, plain water intake; Healthy: none of the specified chronic health conditions (obesity, diabetes, hypertension, metabolic syndrome, cancer or evidence of any disorder of the liver, digestive tract, lung, kidney, or cardiovascular system); CHC: One or more chronic health condition; Hydrated: Serum tonicity ≥285 and ≤ 294 **AND** specific urine gravity < 1.013; Model 1: adjusted for antihypertensive medication, exercise, renal solute load, hypertension, kidney disorder, smoking and season of visit; Model 2: adjusted for all variables in Model 1 plus BMI outside of normal range (18.5–25.0).

Control for factors that determine water requirements did not explain away the significant difference in reported level of water intake between the Healthy+Hydrated and Healthy+Not Hydrated groups. As can be seen in Appendix 4, the direction, magnitude, and statistical significance of the results did not change when urine creatinine concentration was used to classify hydration instead of specific urine gravity.

### 3.4 Water intake associated with hydration and health

#### 3.4.1 PWI and TWI bivariate distribution

**Figure 2:**
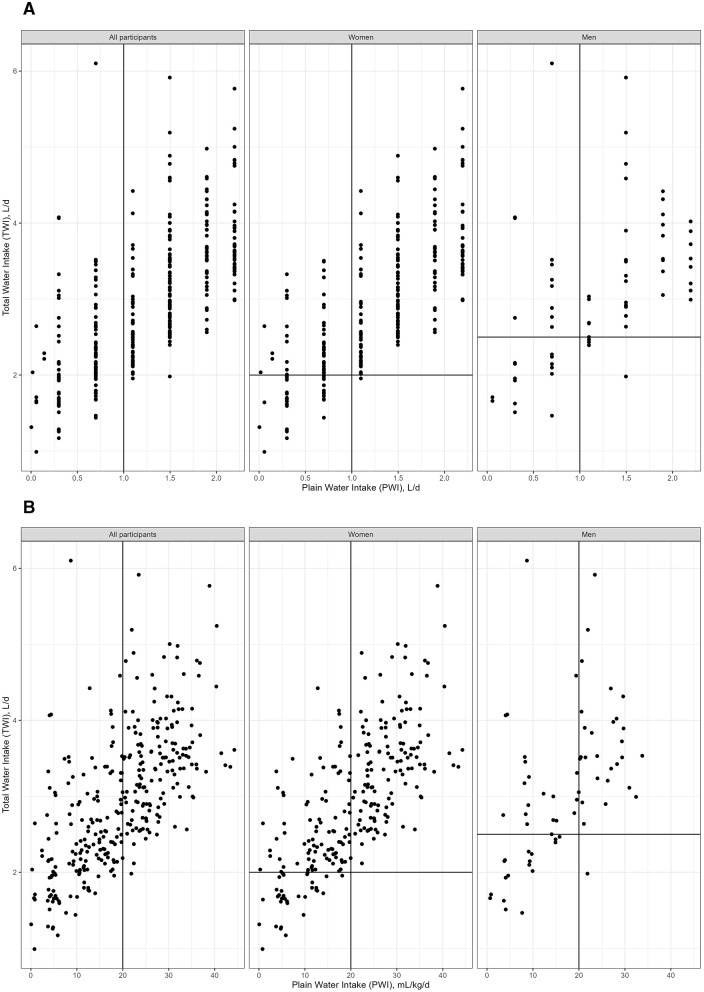
Bivariate distribution of PWI and TWI in the Healthy+Hydrated group. PWI, plain water intake; TWI, total water intake; Healthy: none of the specified chronic health conditions (obesity, diabetes, hypertension, metabolic syndrome, cancer or evidence of any disorder of the liver, digestive tract, lung, kidney, or cardiovascular system); CHC: One or more chronic health condition; Hydrated: Serum tonicity ≥285 and ≤ 294 AND specific urine gravity < 1.013; to make Figure 2 more readable 16 TWI values above 125 mL/kg/d were removed. The associated PWI for all removed points was between 1.4 and 1.9 L/d. **(A)** figure has PWI in L/d. **(B)** figure has PWI in mL/kg/d.

Figure 2 describes the bivariate distribution of PWI and TWI in the Healthy+Hydrated group. PWI and TWI covaried such that almost all participants who reported PWI over 1 L/d had TWI over 2 L/d. Almost all participants who reported over 20 mL/kg PWI, also reported a TWI at or above the European Food Safety Authority (EFSA) AI reference value for TWI (2.5 L/d for men; 2.0 L/d for women). None of the Healthy+Hydrated participants reported zero PWI. Overall, for the Healthy+Hydrated group, PWI accounted for 41% of TWI. For 10% of the Healthy+Hydrated subgroup, PWI accounted for over 60% of TWI. Appendix 5 describes the bivariate distribution of PWI and TWI for those participants who obtained more than 60% of their TWI from PWI.

#### 3.4.2 Median PWI and TWI

Appendix 6 and Table 3 describe the gender-specific univariate PWI and TWI distributions for the Healthy+Hydrated group. For women and men, respectively, the median PWI was 1.5 L/d (22 mL/kg/d) and 1.3 L/d (17 mL/kg/d). The median TWI was 2.9 L/d (45 mL/kg/d) and 3.0 L/d (40 mL/kg/d), respectively. Half of the Healthy+Hydrated group reported over 1 L/d PWI. Almost all (97%) of the Healthy+Hydrated group reported a PWI under 2.2 L/d. For the Healthy+Hydrated participants who obtained the majority of TWI from PWI, PWI ranged between 20 and 45 mL/kg/d.

**Table 3:** Gender-specific percentiles of the TWI and PWI distributions by chronic health condition and hydration classification.

**Water intake**	**Percentile**	**Healthy**+**Hydrated**		**Healthy+Not Hydrated**	**CHC+Hydrated**	**CHC+Not Hydrated**
		**All**	>**60% TWI from PWI**	**All**	**All**	**All**
		**(*****n*** = **251)**	**(*****n*** = **26)**	**(*****n*** = **1,083)**	**(*****n*** = **192)**	**(*****n*** = **1,462)**
**Women**						
Total water intake, mL/kg/d	3	22.9	36.7	21.4	20.1	17.8
	25	35.6	43.8	33.7	30.0	29.1
	50	45.4	49.3	42.2	39.3	36.9
	75	56.0	56.4	52.5	50.3	47.1
	97	79.2	68.7	72.9	65.7	71.4
Total water intake, L/d	3	1.6	2.4	1.4	1.5	1.4
	25	2.3	2.9	2.2	2.2	2.2
	50	2.9	3.4	2.1	2.9	2.7
	75	3.5	3.5	3.3	3.3	3.3
	97	4.8	3.6	4.5	4.7	4.6
Plain water intake, mL/kg/d	3	3.8	22.6	1.0	3.3	1.4
	25	12.5	29.8	11	10.9	10.1
	50	21.6	34.1	17.7	17.8	15.5
	75	28.0	35.8	25.8	25.5	22.3
	97	37.5	43.6	37.3	35.9	35.9
Plain water intake, L/d	3	0.3	1.5	0.1	0.3	0.1
	25	0.7	1.9	0.7	0.7	0.7
	50	1.5	2.2	1.1	1.1	1.1
	75	1.9	2.2	1.5	1.9	1.5
	97	2.2	2.2	2.2	2.2	2.2
**Water intake**	**Percentile**	**Healthy**+**Hydrated**		**Healthy**+**Not Hydrated**	**CHC**+**Hydrated**	**CHC**+**Not Hydrated**
		**All**	>**60% TWI from PWI**	**All**	**All**	**All**
		**(*****n*** = **66)**	**(*****n*** = **7)**	**(*****n*** = **759)**	**(*****n*** = **118)**	**(*****n*** = **1,886)**
**Men**						
Total water intake, mL/kg/d	3	19.9	29.5	18.3	17.6	14.6
	25	32.2	35.2	28 = 9.0	27.0	24.4
	50	39.9	43.9	35.4	34.3	31.1
	75	50.2	44.2	44.1	44.1	39.3
	97	75.9	52.5	64.6	73.3	60.9
Total water intake, L/d	3	1.6	2.16	1.5	1.5	1.3
	25	2.4	3.0	2.4	2.4	2.2
	50	3.0	3.1	2.8	3.0	2.7
	75	3.5	3.3	3.4	3.6	3.4
	97	5.2	3.5	4.8	5.9	5.1
Plain water intake, mL/kg/d	3	3.4	20.3	0.8	0.9	0.2
	25	8.7	24.1	7.9	7.9	4.0
	50	17.4	28.3	11.1	11.9	9.2
	75	23.3	31.7	18.5	18.0	15.6
	97	31.0	33.5	29.1	30.3	26.5
Plain water intake, L/d	3	0.3	1.6	0.1	0.1	0.0
	25	0.7	2.1	0.7	0.7	0.3
	50	1.3	2.2	0.7	1.1	0.7
	75	1.8	2.2	1.5	1.5	1.5
	97	2.2	2.2	2.2	2.2	2.2

Percentile: Percentiles of the TWI or PWI distributions; TWI: Total water intake; PWI: Plain water intake; Hydrated: Serum tonicity ≥285 and ≤ 294 AND specific urine gravity < 1.013; CHC: One or more chronic health condition; Healthy: none of the specified chronic health conditions (obesity, diabetes, hypertension, metabolic syndrome, cancer or evidence of any disorder of the liver, digestive tract, lung, kidney, or cardiovascular system).

## 4 Discussion

This analysis was motivated by longstanding confusion about how much plain water the average person should drink and persistent gaps in “objective evidence” about plain water intake (3). The study contributes observational data about the PWI, hydration, and health of free-living adults in metropolitan Salzburg, Austria. These data can inform policymakers about the dose(s) of water intake that are relevant, feasible, and associated with hydration and health under local daily life conditions. When aligned with data from randomized trials that test the same dose(s), these data can facilitate generalization of causal effects from controlled experimental conditions to conditions of daily life in Austria.

The present study described PWI associated with hydration and health, at ages 40–70 years, when chronic health problems manifest. It explored the PWI of Salzburg residents, given their usual water intake from foods and other beverages, as well as the special case when PWI is the primary means of meeting water intake requirements.

### 4.1 Data from free-living adults who are healthy and hydrated

AI reference values for TWI are set based on median intakes of healthy people in population-representative data (6, 7). Current water intake recommendations in Europe and the U.S. define “healthy” only in terms of acute health, excluding chronic health criteria. Consistent with the high prevalence of CHC in Austria (22), over half of the Paracelsus 10,000 study population had at least one CHC, even though none of the participants were acutely ill on when data were collected. Consistent with other population representative datasets (23), a majority of study participants did not meet hydration criteria.

Study groups that had one or more CHC and/or did not meet hydration criteria were significantly more likely to report lower water intake than the Healthy+Hydrated group. The median PWI for women (1.1 L/d) and men (0.7 L/d) in the overall sample were lower than the median PWI for women (1.5 L/d) and men (1.3 L/d) in the Healthy+Hydrated group. Given that only 15% of women and 7% of men in the Paracelsus 10,000 study population met both health and hydration criteria, results from this group could be masked by results for the greater majority of participants with CHC. Water intake requirements for preventing incident CHC may be significantly underestimated if derived from study populations with CHC.

In the Paracelsus 10,000 study population, the median TWI for Healthy+Hydrated women and men (2.9 and 3.0 L/d, respectively) were higher than the EFSA sex-specific AI for TWI of 2.0 and 2.5 L/d (6) and the German Society for Nutrition TWI recommendation of 2,270–2,360 mL/d (24).

Lower water intake in groups that did not meet hydration criteria (Healthy+Not Hydrated and CHC+Not Hydrated) did not appear to be attributable to lower water requirements. The difference between the hydrated and not hydrated groups was not explained away by control for determinants of water requirements. The analyses suggest need for clinical trials to determine if water intake was insufficient relative to requirements for the Not Hydrated groups.

Lower water intake in groups that met hydration criteria, but not chronic health criteria, appeared attributable to between-group differences in water requirements. Control for factors, including physical activity and body size, explained away the difference between the CHC+Hydrated and Healthy+Hydrated groups. Trending lower physical activity and significantly higher body weight could explain the observed lower water intake per kg body weight for the CHC+Hydrated group compared to the Healthy+Hydrated group.

### 4.2 PWI in relation to TWI under conditions of daily life

Public health messages about water imply that PWI and TWI are interchangeable. According to the NAM in the United States, for example (7): “It is possible to meet the AI for total water by consuming little or no plain water, but instead by consuming a mixed diet (including fruits and vegetables, most of which are over 90 percent water by weight; meat, fish, and poultry, which contain about 60–70 percent water by weight; and other beverages, such as fruit juices and milk….).”

Randomized experiments, nevertheless, show differences in water absorption and short-term metabolism depending on the source of water. Unlike plain drinking water, which has an osmolality below 20 mOsm/kg, most retail beverages and foods have an osmolality above that of normal plasma osmolality (>285 mOsm/kg) (20). Whereas plain water moves into body water compartments within seconds (25), hypertonic fluids *shift body water out of cells* into the gut lumen to dilute the beverage before it is absorbed (26). Drinking water instead of caloric beverages (e.g., sugar-sweetened beverages, milks, and juices) with meals consistently results in lower total energy intake, lower insulin levels, and greater postprandial fat oxidation (27). Unlike drinking water, hypertonic beverages cause body water retention (28) *by creating extracellular hypertonicity*, which triggers the kidneys to concentrate urine and reduce urine volume to normalize extracellular tonicity and cell volume. Higher plasma osmolality is associated with altered sympathetic nerve discharge (29), heart rate, energy expenditure (30), macronutrient metabolism, and tissue and organ function (31, 32). Studies suggesting that beverages hydrate equally, regardless of osmolality ignore these many differences [e.g., see Ref. (33)].

Noting that TBW deficit and death result when TWI is primarily composed of water from hypertonic sources, such as overly concentrated infant formula or sea water (in desperate circumstances) (34, 35), the composition of TWI clearly matters. In the present population-based dataset, *none* of the Healthy+Hydrated men or women reported zero PWI. If individuals cannot achieve hydration and long-term health with zero PWI under conditions of daily life, public health messages that imply no requirement for PWI may need to be revised.

At the population level, underestimation of the AI for PWI may conceivably increase CHC risk. An increasing body of observational literature links suboptimal PWI or underhydration with increased risk of CHC incidence, progression, and mortality (8, 36). In longitudinal data, greater intake of plain drinking water, either absolute or relative (instead of caloric beverages), is associated with lower risk of obesity, non-alcoholic fatty liver disease, and incident diabetes (27, 37, 38).

### 4.3 How much PWI does the average adult need for hydration and long-term health?

Results of the present analysis suggest that, for the average adult living in Salzburg, Austria, where *ad-libitum* foods and beverages provide 60% of TWI, hydration and health at ages 40–70 is associated with drinking at least 1 L/d (16 mL/kg/d), but less than 2.2 L/d plain water. Half of the Healthy+Hydrated group reported over 1 L/d PWI. The PWI intake for 97% of the Healthy+Hydrated was below 2.2 L/d. PWI of at least 1.0 L/d is consistent with the French Programme National Nutrition Sante recommendations for adults to consume between 1.0 and 1.5 L/d plain water (39). PWI of at least 1.0 L/d is consistent with the Austrian Agency for Health and Food Safety recommendation of “at least 1.5 L of liquid (beverages) every day” (40). Randomized clinical trials are needed to check for effects of 1.0, 1.5, and 2.0 L/d PWI on hydration and long-term health parameters, in people living in Austria who usually consume 60% of TWI from foods and beverages other than plain water.

Results from a few clinical studies in the U.S. suggest improved hydration and reduced CHC risk associated with increasing PWI by 1.0–1.5 L/d over 4–8 weeks. The sustained higher PWI was associated with a significant shift in metabolism away from aestivation and Warburg-type patterns in healthy normal weight adult men (41). It was associated with greater weight loss in women with overweight or obesity on weight loss diets (42).

For the subset of Healthy+Hydrated individuals who obtained over 60% of their TWI from PWI, PWI ranged as high as 20–45 mL/kg, a level which is on par with the minimum TWI requirement to replace water losses estimated for a 70 kg human in a temperate zone by the Tropical Agriculture Association (42.9 mL/kg/d) (43) and the TWI requirement estimated for a 70 kg adult with a 3,000 kcal/d diet and a urine specific gravity of 1.020 g/mL (40 mL/kg/d) or 1.015 g/mL (45 mL/kg/d) (6).

### 4.4 Limitations

Only cross-sectional data from people ages 40–70 were available for the present analysis. Interpretation of the results and inferences about health over the life course, through to ages 40–70, depend on the assumption that individuals, who did not have chronic health conditions when they were measured for the Paracelsus 10,000 study, *never* had a chronic health condition. Longitudinal studies are needed to confirm effects of PWI over the life course.

Although the Paracelsus 10,000 study recruited participants by random sampling from gender- and age group-specific lists of residents in the metropolitan Salzburg area, results for the health- and hydration-stratified groups may be vulnerable to selection bias, due to small numbers. Only 251 women and 66 men, small fractions of the total study population, met the Healthy+Hydrated criteria.

PWI estimates in this analysis were derived from responses to the EPIC food frequency questionnaire (FFQ), which asks participants to estimate the usual number of discrete glasses of water, consumed over the past 12 months, and has an upper bound of 11 possible glasses. In addition to potential recall errors related to the year-long period, the median TWI in this study can be expected to underestimate the true TWI, and overestimate the fraction of TWI that is PWI, because FFQs systematically underestimate intakes by not capturing every food and beverage consumed. The fixed upper bound, while not affecting the estimated median intakes, provides no information about maximum water intake. PWI was at or above the upper bound for 8% of the study population. Studies involving more complete TWI and PWI assessment are needed to determine AI volumes.

The results of hydration prevalence studies depend on the choice of hydration biomarker (44). As there is no one gold standard hydration biomarker (45), various hydration biomarkers are in use. The EFSA defines hydration in terms of a “desirable urine osmolality” of 500 mOsm/kg which leaves a safe margin of free water reserve (6). The U.S. and Canadian NAM (7) define hydration in terms of serum osmolality within the normal range. This analysis classified hydration with respect to serum and urine measures, in alignment with analyses of population-representative NHANES data from the U.S (23).

Serum tonicity was estimated using an equation proposed by Matz (14), which is equivalent to an equation for serum osmolarity, minus solute which freely cross cell membranes and are not osmotically effective (in this case, blood urea nitrogen). The equation for serum osmolarity was validated by Heavens et al. [equation #26 (46)] and found to have 100% of calculated values within 10 mmol of measured osmolality and a low mean absolute error of 1.9. Equations for tonicity are vulnerable to errors related to osmotically ineffective *solute that become osmotically effective* with aging or CHC, due to insulin resistance and altered solute transport across cell membranes. If osmotically effective solute concentrations were underestimated in this study, due to use of the equation by Matz, misclassification bias might be expected to disproportionately affect the CHC+Hydrated group, and bias differences between the CHC+Hydrated vs. Healthy+Hydrated. The *tonicity-*based hydration classification and results of the present analysis would not be changed by subtraction of blood urea nitrogen from alternative validated equations for serum osmolarity, which are recommended for use in healthy adults (46) and older adults (47) (e.g., the Worthley equation #6 in Ref. (46): (2 × sodium)+glucose+ blood urea nitrogen) or Khajuria and Krahn equation #34 in reference (46): (1.86 × (Na+K) + (1.15 × glucose)+urea+14)). In sensitivity analyses that replace the Matz equation with the Worthley and Khajuria and Krahn equations, the statistical significance and direction of reported effects remain unchanged (data not shown).

Urine osmolality is informative about free water deficit, i.e., the need for plain water, but inference depends on urine concentration ability. Individuals, who cannot concentrate urine, produce dilute urine, despite elevated serum osmolality, and lose body water instead of retaining it, when necessary. Urine concentrating ability, which declines with age (48), is thus a source of misclassification error for the urine osmolality biomarker in middle aged or older adults. Serum solute concentrations, which are widely recognized biomarkers of TBW deficit (7), are relatively insensitive to mild TBW deficit, because of homeostatic mechanisms, including vasopressin release and urine concentration, which compensate for TBW deficit by shifting body fluid to protect blood volume (49). The use of both urine and serum biomarkers, together, addresses each respective limitation and makes a non-trivial difference to the proportion of people classified as hydrated in population-representative data (23).

The spot urine and blood measures available for this analysis only classify people with respect to hydration at a moment in time, which may not reflect 24-h or usual hydration, though concentrated morning urine osmolality in adults aged 23–62 years has approximately 80% sensitivity and specificity for usual underhydration (50) and there is little intraindividual variation in serum sodium over long periods (10 years) (36).

In this analysis of age group-balanced data, population-representative weights were not applied. To define gender-specific AI for PWI in Salzburg, using the median water intake approach followed by the NAM (7), weighted population-representative median PWI for men and women would be needed.

Water intake recommendations aim to represent the amount of water that an average person needs to replace water losses (6, 7). Given the many factors that determine water losses, the recommendations do not cover the needs of every individual in the population. Individual requirements vary widely because of variation in physical activity, ambient temperature, clothing, altitude, diet, medications, and health condition (3, 7). The results of this study pertain to men and women ages 40–70, who live in Salzburg, Austria. Observational data are needed from other periods and communities to check if the findings generalize to other climates and demographic groups.

This analysis pursued information about the level of PWI and TWI of people who are healthy. The participants' health condition was classified based on laboratory tests and a range of clinical measures but may exclude some conditions, such as dementia and arthritis, for which reliable measures were not collected. Misclassification of people with unobserved or pre-clinical CHC in the Healthy group is a potential source of error. Studies to determine the AI for water to prevent incident conditions must exclude people with the condition at baseline.

Public health recommendations are needed for everyone, including people with chronic health conditions, who make up the majority of free-living adults over age 50. Cross-sectional data from the present study, which do not discern between water intakes that are cause vs. consequence of CHC, cannot address this purpose. Longitudinal data are needed about levels of PWI and TWI to recommend for CHC treatment.

In 2002, Valtin (2) described “seemingly ubiquitous admonition to ‘drink at least eight 8-oz glasses of water a day”' as well as skepticism about the “8 × 8” recommendation, in the lay press, and called for evidence to resolve the confusion. Valtin noted, “Despite an extensive search of the literature […], I have found no scientific reports concluding that we all must ‘drink at least eight glasses of water a day”'. Two decades later, in 2022, Yamada et al. (3) call attention to persistent confusion about drinking water with the comment that “the common suggestion to drink eight 8-ounce glasses of water per day (~2 L) is not backed up by objective evidence.” The present study offers observational evidence about PWI in Austria. If/when clinical trials test for effects of PWI, given background TWI, the results signal potential to and develop drinking water- specific recommendations such as “drink at least 1 L of plain water per day as part of your usual diet.”

## 5 Conclusions

This study used observational, population-based data to estimate *how much PWI* is associated with hydration and health, under conditions of daily life in Austria. The results suggest that the amount is *not zero*; Hydration and health appeared associated with *some minimum amount* of plain drinking water that exceeds 1 L/d as part of total water intake. The data warrant randomized controlled trials in Austria to test for causal effects of PWI in the range of 1–2 L/d, given usual *ad-libitum* diet, and effects of 20–45 mL/kg/d PWI, if PWI provides the majority of TWI. Further work is needed to gather community-specific, observational and clinical evidence about plain drinking water to develop population-level recommendations about how much plain water to drink for long-term health.

## Data Availability

The data analyzed in this study is subject to the following licenses/restrictions: Permission to access and analyze Paracelsus 10,000 study data is restricted. Requests to access these datasets should be directed to: e.trinka@salk.at.
