# Supplementary material for: Hydration and health at ages 40–70 years in Salzburg Austria is associated with a median total water intake over 40 mL/kg including at least 1 L/d plain drinking water
Source: Front Public Health. 2025 Nov 7;13:1668981. doi: 10.3389/fpubh.2025.1668981 (PMC12634361; doi:10.3389/fpubh.2025.1668981)
Supplement: Supplementary file 1 [file Supplementary_file_1.zip › Appendix figures and tables-list.docx]

# Supplementary figure titles and legends and tables

**Appendix Figure 1**. Strengthening the Reporting of Observational Studies in Epidemiology (STROBE) flow diagram

The number of people who were eligible, excluded, invited, and consented to participate in the Paracelsus 10000 study, who completed baseline data collection and were excluded or included in the present analysis.

**Appendix Figure 2**. Estimated mean daily total water intake for the Paracelsus 10,000 study cohort by source of water intake and gender

To make the plot easier to read, 140 values above 3 L/d are not shown; Of these, 100 values were from alcoholic beverages, 20 were from sugar sweetened beverages and 20 were from other beverages.

**Appendix Table 3**. Chronic health parameters of participants in the Paracelsus 10,000 study

**Appendix Table 4**. Relative risk of lower water intake associated with chronic health condition and hydration classification defined in terms of urine creatinine concentration instead of specific urine gravity.

**Appendix Figure 5**. Bivariate distribution of plain water intake (PWI) and total water intake (TWI) for participants in the Healthy+Hydrated group who obtained more than 60% of their TWI from PWI

Healthy: None of the specified chronic health conditions (obesity, diabetes, hypertension, metabolic syndrome, cancer or evidence of any disorder of the liver, digestive tract, lung, kidney, or cardiovascular system); Hydrated: Serum tonicity ≥ 285 and ≤ 294 AND specific urine gravity < 1.013.

**Appendix Figure 6**. Gender-specific univariate distributions of plain water intake and total water intake for participants in the Healthy+Hydrated group

PWI: Plain water intake; TWI: Total water intake; Healthy: None of the specified chronic health conditions (obesity, diabetes, hypertension, metabolic syndrome, cancer or evidence of any disorder of the liver, digestive tract, lung, kidney, or cardiovascular system); Hydrated: Serum tonicity ≥ 285 and ≤ 294 AND specific urine gravity < 1.013. Curves represent probability density. Horizontal box plots describe the minimum value (left whisker), median value (vertical line in bar), and maximum value (right whisker) of the distribution, excluding outliers (dots).
