# Supplementary material for: Hydration and health at ages 40–70 years in Salzburg Austria is associated with a median total water intake over 40 mL/kg including at least 1 L/d plain drinking water
Source: Front Public Health. 2025 Nov 7;13:1668981. doi: 10.3389/fpubh.2025.1668981 (PMC12634361; doi:10.3389/fpubh.2025.1668981)
Supplement: Supplementary file 1 [file Supplementary_file_1.zip › Appendix_3_table.docx]

Appendix 3. Chronic health parameters of participants in the Paracelsus 10,000 study

|  | Women  n=2,988 | | Men  n=2,829 | |
| --- | --- | --- | --- | --- |
|  | n | % of Sample | n | % of Sample |
| Obesity/Underweight | 498 | 17 | 568 | 20 |
| Underweight | 51 | 2 | 7 | 0.3 |
| Obesity | 447 | 15 | 561 | 20 |
| Kidney Disorder | 233 | 8 | 171 | 6 |
| Glomerulonephritis | 3 | 0.1 | 1 | 0.0 |
| eGFR < 60 (mL x 1.73m^2^/min) | 124 | 4 | 69 | 2 |
| Albumin/Creatinine ratio ≥ 30 | 113 | 4 | 114 | 4 |
| Diabetes, Prediabetes or Insulin Resistance | 657 | 22 | 1105 | 39 |
| Type 1 Diabetes | 4 | 0.1 | 2 | 0.1 |
| Type 2 Diabetes | 52 | 2 | 121 | 4 |
| Fasting Plasma Glucose ≥ 110 mg/dL | 137 | 5 | 361 | 13 |
| HOMA-IR ≥ 2.5 | 626 | 21 | 1028 | 36 |
| Antidiabetic Medication | 47 | 2 | 100 | 4 |
| Hypertension | 860 | 29 | 1404 | 50 |
| Systolic Blood Pressure ≥140 (mmHg) | 528 | 18 | 806 | 29 |
| Diastolic Blood Pressure ≥ 90 (mmHg) | 407 | 14 | 906 | 32 |
| Antihypertensive Medication | 395 | 13 | 596 | 21 |
| Cardiovascular Disorder | 81 | 3 | 192 | 7 |
| Coronary Artery Disease | 19 | 1 | 70 | 3 |
| Stroke | 33 | 1 | 52 | 2 |
| Perihperal Arterial Disease | 5 | 0.2 | 12 | 0.4 |
| Abdominal Aortic Aneurysm | 3 | 0.1 | 13 | 1 |
| Atrial Fibrillation | 21 | 1 | 52 | 2 |
| Chronic Heart Failure | 9 | 0.3 | 20 | 1 |
| Stenosis | 2 | 0.0) | 11 | 0.4 |
| Metabolic Syndrome ^a^ | 432 | 15 | 768 | 27 |
| Criterion 1 | 1256 | 42 | 983 | 35 |
| Criterion 2 | 345 | 12 | 771 | 27 |
| Criterion 3 | 256 | 9 | 315 | 11 |
| Criterion 4 | 1306 | 44 | 1941 | 69 |
| Criterion 5 | 442 | 15 | 908 | 32 |
| Cancer | 226 | 8 | 142 | 5 |
| Liver Disorder | 13 | 0.4 | 24 | 1 |
| Cirrhosis | 1 | 0.0 | 8 | 0.3 |
| Chronic Hepatitis | 12 | 0.4 | 18 | 1 |
| Digestive Tract Disorder | 16 | 1 | 18 | 1 |
| Colitis Ulcerosa | 13 | 0.4 | 13 | 1 |
| Crohn's Disease | 3 | 0.1 | 5 | 0.2 |
| Pulmonary Disorder | 237 | 8 | 262 | 9 |
| COPD | 28 | 1 | 45 | 2 |
| Asthma | 186 | 6 | 189 | 7 |
| Pulmonary Embolism | 37 | 1 | 40 | 1 |

^a^ Criterion 1: Abdominal Circumference > 88 cm for women or > 102 cm for men; Criterion 2: Triglycerides levels ≥ 150 mg/dL **OR** on fibrate therapy; Criterion 3: HDL-Cholesterol < 50 mg/dL for women or < 40 mg/dL for men; Criterion 4: Systolic Blood Pressure ≥ 130, Diastolic Blood Pressure ≥ 85 mmHg **OR** on antihypertensive drug therapy; Criterion 5: Fasting Plasma Glucose ≥ 100 mg/dL **OR** on antidiabetic drug therapy.
