# Supplementary material for: Hydration and health at ages 40–70 years in Salzburg Austria is associated with a median total water intake over 40 mL/kg including at least 1 L/d plain drinking water
Source: Front Public Health. 2025 Nov 7;13:1668981. doi: 10.3389/fpubh.2025.1668981 (PMC12634361; doi:10.3389/fpubh.2025.1668981)
Supplement: Supplementary file 1 [file Supplementary_file_1.zip › Appendix_4_table.docx]

Appendix 4. Relative risk of lower water intake associated with chronic health condition and hydration classification defined in terms of urine creatinine concentration instead of specific urine gravity.

|  | Health  condition | Hydration  classification | Level of water intake | | | Relative risk of having lower water intake | | | | | |
| --- | --- | --- | --- | --- | --- | --- | --- | --- | --- | --- | --- |
|  |  |  | Higher | Lower | | Unadjusted | | Model 1 | | Model 2 | |
|  |  |  | n | n | Row% | RR (95%CI) | p-value | RR (95%CI) | p-value | RR (95%CI) | p-value |
| Women | Healthy | Hydrated | 120 | 175 | 40.7 | 1.0 | - | 1.0 | - | 1.0 | - |
|  | Healthy | Not Hydrated | 642 | 697 | 52.1 | 1.18 (1.02, 1.37) | .030 | 1.18 (1.02, 1.37) | .028 | 1.18 (1.02, 1.37) | .029 |
|  | CHC | Hydrated | 124 | 109 | 53.2 | 1.31 (1.09, 1.57) | .004 | 1.23 (1.02, 1.48) | .029 | 1.14 (0.95, 1.37) | .167 |
|  | CHC | Not Hydrated | 1151 | 703 | 62.1 | 1.53 (1.32, 1.76) | <.001 | 1.44 (1.24, 1.67) | <.001 | 1.32 (1.14, 1.54) | <.001 |
| Men | Healthy | Hydrated | 38 | 33 | 53.5 | 1.0 | - | 1.0 | - | 1.0 | - |
|  | Healthy | Not Hydrated | 631 | 270 | 70.0 | 1.31 (1.05, 1.63) | .017 | 1.31 (1.06, 1.63) | .013 | 1.31 (1.06, 1.63) | .013 |
|  | CHC | Hydrated | 96 | 39 | 71.1 | 1.33 (1.04, 1.69) | .021 | 1.32 (1.04, 1.67) | .023 | 1.28 (1.01, 1.63) | .039 |
|  | CHC | Not Hydrated | 1897 | 472 | 80.1 | 1.50 (1.20, 1.86) | <.001 | 1.48 (1.20, 1.84) | <.001 | 1.43 (1.16, 1.77) | .001 |

Lower water intake: TWI < 45 mL/kg **AND** PWI < 20 mL/kg; Higher water intake: TWI ≥ 45 mL/kg **OR** PWI ≥ 20 mL/kg; Healthy: None of the specified chronic health conditions (obesity, diabetes, hypertension, metabolic syndrome, cancer or evidence of any disorder of the liver, digestive tract, lung, kidney, or cardiovascular system); CHC: One or more chronic health condition; Hydrated: Serum tonicity ≥ 285 and ≤ 294 **AND** urine creatinine < 89; Model 1: adjusted for antihypertensive medication, exercise, renal solute load, hypertension, kidney disorder, smoking and season of visit; Model 2: adjusted for all variables in model 1 plus BMI outside of normal range (18.5 – 25.0).
