# Supplementary figures and images for: Hydration and health at ages 40–70 years in Salzburg Austria is associated with a median total water intake over 40 mL/kg including at least 1 L/d plain drinking water
Source: Front Public Health. 2025 Nov 7;13:1668981. doi: 10.3389/fpubh.2025.1668981 (PMC12634361; doi:10.3389/fpubh.2025.1668981)

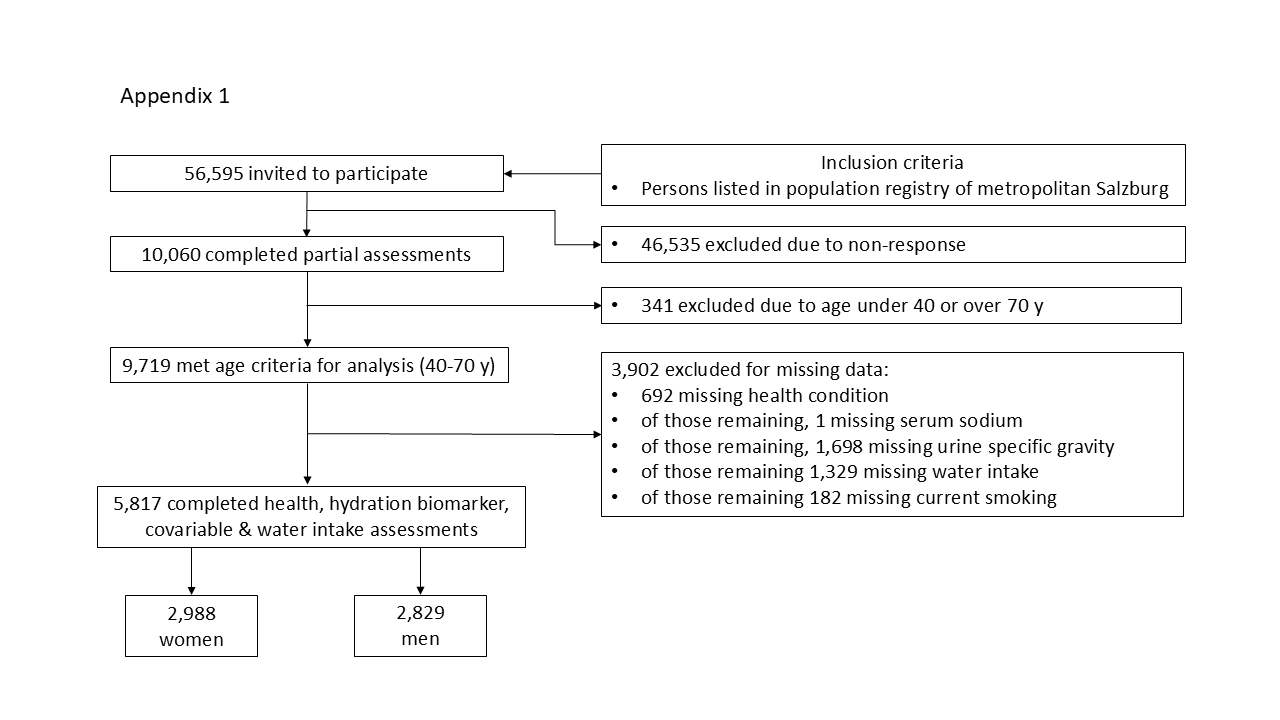

Supplement: Supplementary file 1 [file Supplementary_file_1.zip › Appendix_1_figure.tif]

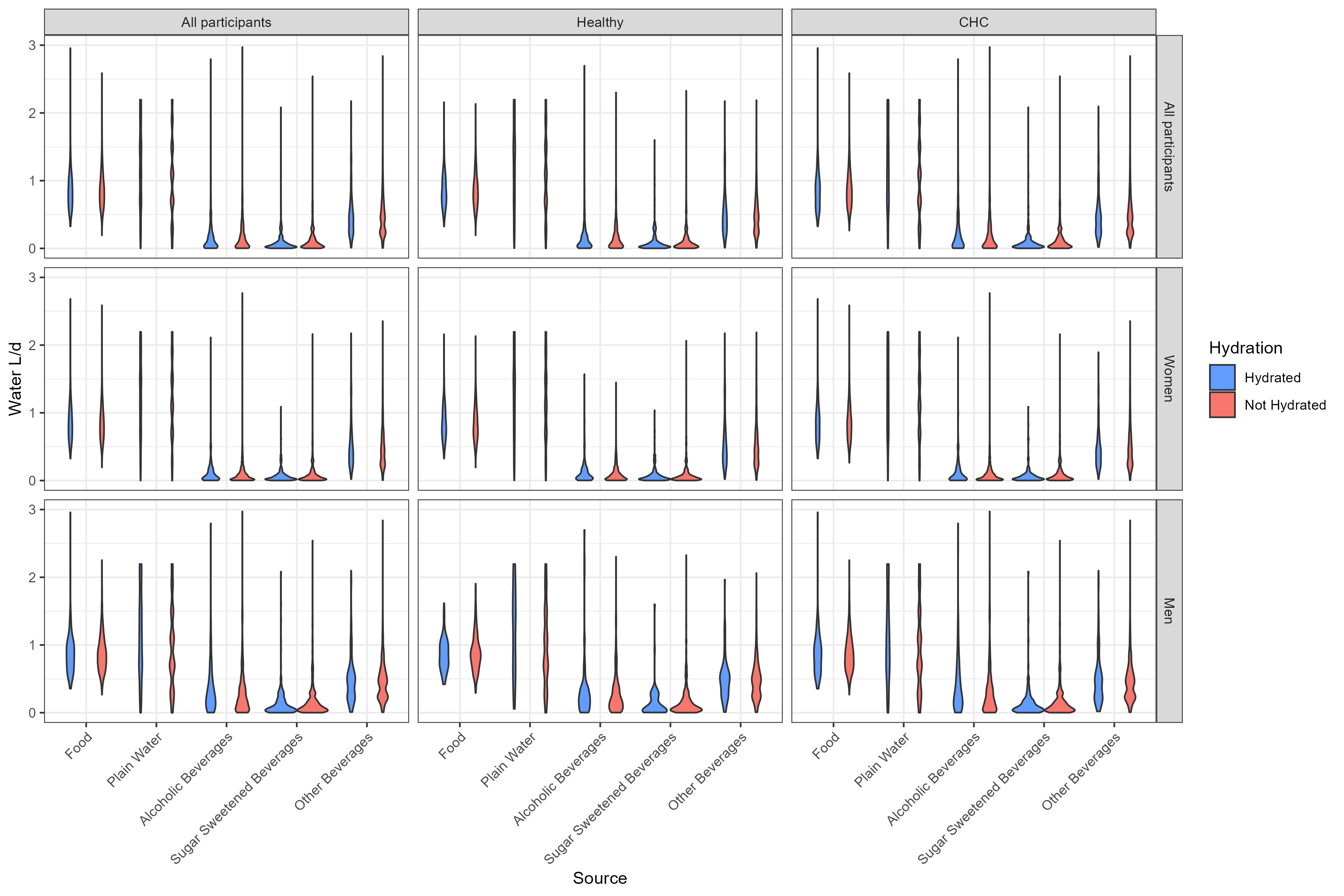

Supplement: Supplementary file 1 [file Supplementary_file_1.zip › Appendix_2_figure.tiff]

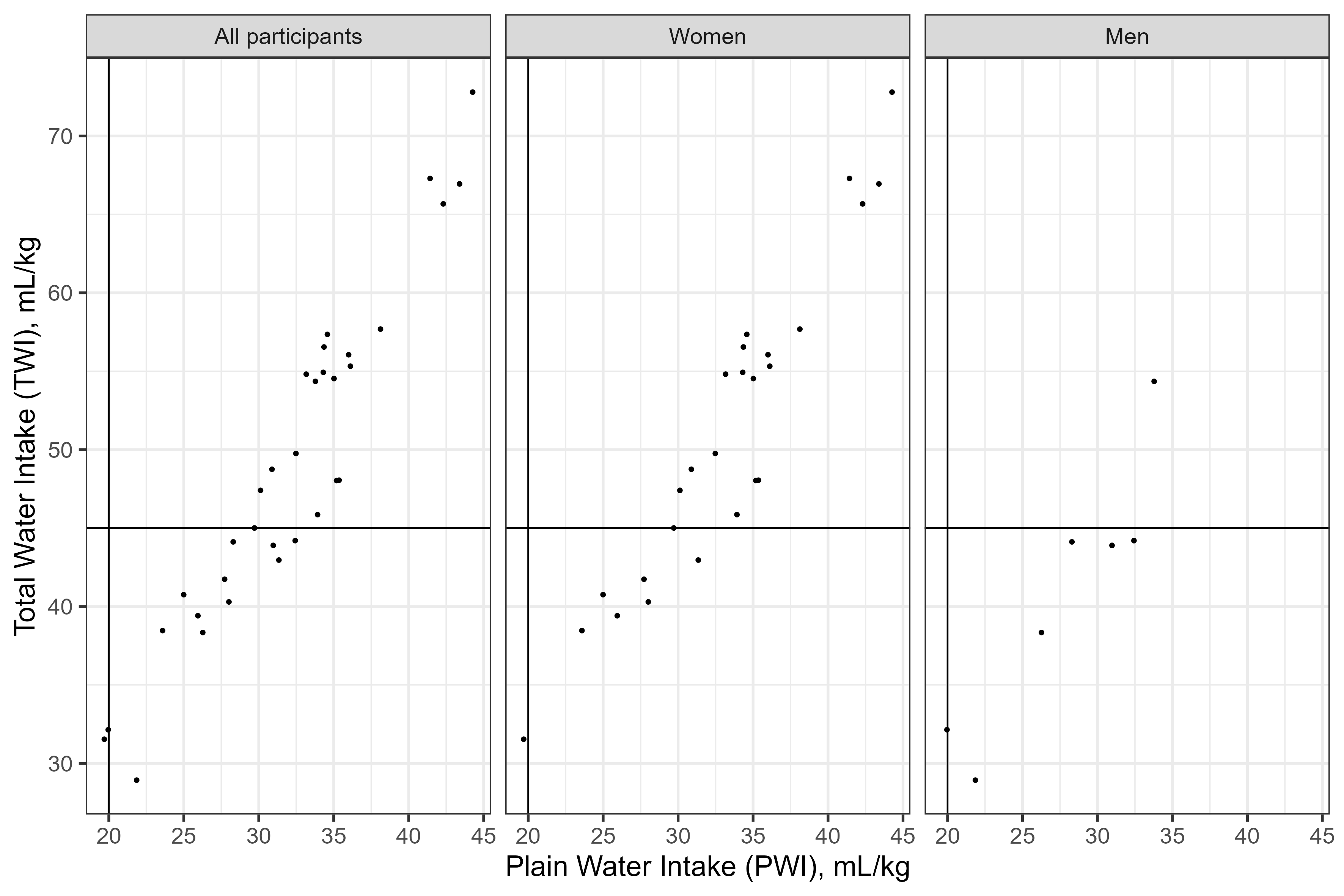

Supplement: Supplementary file 1 [file Supplementary_file_1.zip › Appendix_5_figure.tiff]

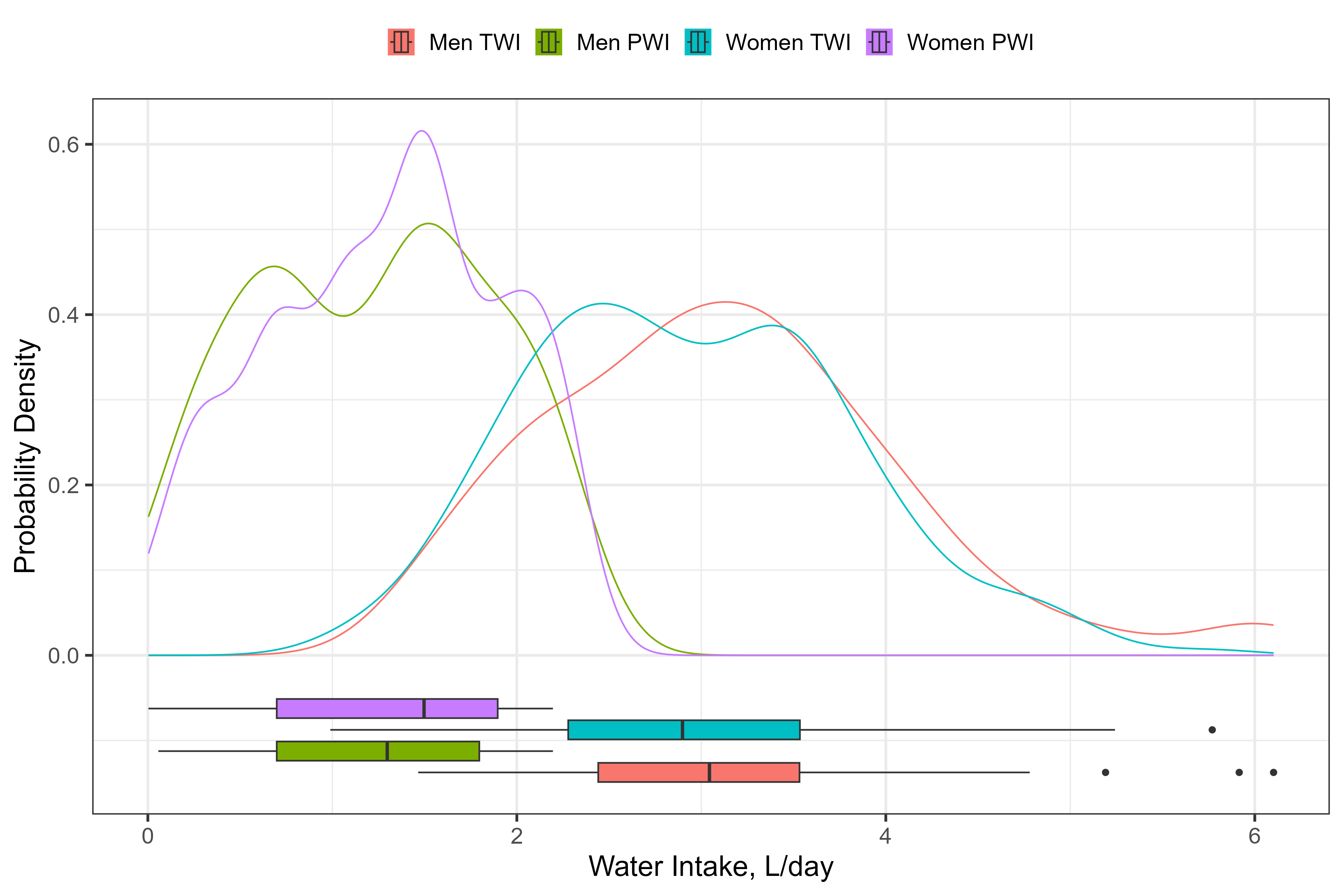

Supplement: Supplementary file 1 [file Supplementary_file_1.zip › Appendix_6_figure.tiff]
